# Synthesis of S-Layer Conjugates and Evaluation of Their Modifiability as a Tool for the Functionalization and Patterning of Technical Surfaces

**DOI:** 10.3390/molecules20069847

**Published:** 2015-05-27

**Authors:** Ulrike Weinert, Katrin Pollmann, Astrid Barkleit, Manja Vogel, Tobias Günther, Johannes Raff

**Affiliations:** 1 Helmholtz-Zentrum Dresden-Rossendorf, Helmholtz-Institute Freiberg for Resource Technology, Halsbruecker Strasse 34, 09533 Freiberg, Germany; E-Mails: u.weinert@hzdr.de (U.W.); m.vogel@hzdr.de (M.V.); t.guenther@hzdr.de (T.G.); j.raff@hzdr.de (J.R.); 2 Helmholtz-Zentrum Dresden-Rossendorf, Institute of Resource Ecology, Bautzner Landstr. 400, 01328 Dresden, Germany; E-Mail: a.barkleit@hzdr.de

**Keywords:** EDC, modified surface-layer proteins, potentiometric titration, modification rate, bio-functionalization of surfaces, chemical modification, immobilization

## Abstract

Chemical functional groups of surface layer (S-layer) proteins were chemically modified in order to evaluate the potential of S-layer proteins for the introduction of functional molecules. S-layer proteins are structure proteins that self-assemble into regular arrays on surfaces. One general feature of S-layer proteins is their high amount of carboxylic and amino groups. These groups are potential targets for linking functional molecules, thus producing reactive surfaces. In this work, these groups were conjugated with the amino acid tryptophan. In another approach, SH-groups were chemically inserted in order to extend the spectrum of modifiable groups. The amount of modifiable carboxylic groups was further evaluated by potentiometric titration in order to evaluate the potential efficiency of S-layer proteins to work as matrix for bioconjugations. The results proved that S-layer proteins can work as effective matrices for the conjugation of different molecules. The advantage of using chemical modification methods over genetic methods lies in its versatile usage enabling the attachment of biomolecules, as well as fluorescent dyes and inorganic molecules. Together with their self-assembling properties, S-layer proteins are suitable as targets for bioconjugates, thus enabling a nanostructuring and bio-functionalization of surfaces, which can be used for different applications like biosensors, filter materials, or (bio)catalytic surfaces.

## 1. Introduction

Surface-layer (S-layer) proteins represent the outermost cell envelope of numerous bacteria and all archaea (recently reviewed in [1]). They cover the whole cell in a regularly arranged grid structure, which exhibits different symmetries like p2, p4, or p6 [2]. As the outermost cell envelope, their functions are quite diverse, such as acting as a boundary to the environment or a binding matrix for exoenzymes [1,3,4,5,6,7,8,9,10,11]. S-layers are comprised principally of one or several protein or glycoprotein monomers with a molecular weight ranging from 40 to 200 kDa. After isolation of the proteins from their host cells, they are able to spontaneously self-assemble in suspension, at interfaces, or on surfaces, as tube-like or planar structures, herein after referred to as “polymers”. These properties make S-layer proteins quite interesting for applications in biotechnology and material science. S-layer proteins can be used to cover numerous technical surfaces, thus enabling the introduction of diverse functionalities like sorptive or catalytic properties [11,12], or work as binding matrices for enzymes and fluorescent dyes [13,14,15]. For these approaches, the chemical composition of the proteins is of great interest. In contrast to archaeal S-layer proteins, those found in bacteria usually do not contain sulfhydryl (SH)-groups, but instead possess modifiable COOH-, NH_2_-, and OH-groups in relatively high amounts ranging from 10–15 mol % per functional group [14,16]. Because of this, the isoelectric points of most S-layer proteins are slightly acidic. These functional groups can be easily modified chemically with 1-ethyl-3-(3′-dimethylaminopropyl)carbodiimide (EDC) or other conventional crosslinkers. Previous work has chemically linked different enzymes with S-layer proteins with the crosslinker EDC as coupling agent [13]. The modification rate was claimed with 1 enzyme per S-layer unit cells (p6 symmetry). Other studies have covalently bound human IgG antibodies [17] or the allergen Bet v1 to S-layer proteins [18]. In these studies, the location as well as the amount of the bound molecules was reproducible. In a more recent work, S-layer proteins were modified with two different fluorescent dyes that are able to perform a Foerster resonance energy transfer (FRET) [15]. The fluorescent dyes were also bound to the S-layer proteins by EDC. Although these methods introduce random modifications of COOH-groups, the enzymes as well as the fluorescence dyes are regularly attached to the S-layer due to the regular biochemical structure of the protein and the regular nanostructure of the lattice. In case of the enzymes, they can be attached to the S-layer protein pores [13]. In case of the FRET pair, the fluorescent dyes are attached at a distance of roughly 6 nm, which ensures a FRET with a high efficiency. This fact also indicates there exists a highly arranged order of the fluorescent dyes on the S-layer proteins. Additionally, the FRET experiments demonstrated that the modification of S-layer polymers did not disturb their structure, whereas modified monomers would lose their self-assembly properties.

The present work systematically investigates the modification rate of the S-layer protein SlfB from a natural bacterial isolate, namely *Lysinibacillus sphaericus* JG-A12. This strain was isolated from a uranium mining waste pile near Johanngeorgenstadt (JG) in Saxony, Germany. The number of modifiable functional groups of this S-layer protein was determined by potentiometric titration and modification of the S-layer protein with the amino acid tryptophan. In order to introduce additional sulfhydryl groups to the protein, *S*-acetyl residues were coupled to NH_2_-groups of the protein. The results proved the suitability of S-layer proteins as binding matrix for the attachment of different functional biomolecules and inorganic molecules. These experiments are the first steps in evaluating the capability of S-layer proteins to introduce functional groups in high density and determining the amount of potentially available groups. Notably, modification influences self-assembling properties of the S-layers, whereas self-assembly on surfaces prior to modification further reduces the amount of available groups. These issues are discussed here within this manuscript. In combination with their ability to crystallize in monolayers on surfaces, S-layer proteins are attractive for the bio-functionalization of surfaces. Crucial factors for technical applications of such bioconjugates are a reliable and stable coating of a broad spectrum of different surfaces. The prior modification of the surfaces with PEI as described in this paper meets these requirements. Moreover, the chemical modifications even seemed to stabilize the S-layer lattices. In conclusion, here is presented a novel method that allows an easy and reliable construction of patterned functionalized surfaces.

## 2. Results and Discussion

### 2.1. Quantification of Modifiable Functional Groups of the S-Layer Protein SlfB by Potentiometric Titration

The gene sequence and amino acid composition of SlfB has been previously determined [19]. SlfB with a molecular weight of 126 kDa contains 62 aspartate, 63 glutamate, 88 lysine, 15 arginine, and 37 tyrosine residues. Therefore, a total amount of 125 COOH-groups, 103 NH_2_-groups, and 37 OH-groups are theoretically available for modifications. However, due to protein folding, surface charges, and steric hindrance, it can be expected that only a small amount of these groups is really accessible for linkage with molecules. In this work, potentiometric titration was performed in order to elucidate the amount of modifiable groups, and to evaluate the potential of S-layer proteins for the introduction of functional molecules in high densities. The potentiometric titration was performed as described in Section 3.1. and results are given in the following Table 1. The experiments were done repeatedly with solutions of protein monomers and polymers. The pKa values and site densities of monomers and polymers were almost the same, indicating that in both cases the same amounts of COOH- and NH_2_-groups are theoretically available for modification.

**Table 1:** Determined pKa-values of SlfB and modification rates evaluated by potentiometric titration; * can be assigned to NH_2_- or OH-groups.

pKa-Value	Site Density (mol_functional group_/mol_SlfB_)	Ratio of Functional Group in the Protein	
3.98 ± 0.02	28 ± 1.0	22.4% ± 0.8%	COOH
5.65 ± 0.06	8.5 ± 0.7	6.8% ± 0.6%	COOH
7.76 ± 0.06	12 ± 1.0	11.7% ± 1.0%	NH_2_
9.49 * ± 0.04	57 ± 4.0	55.3% ± 3.9%	NH_2_ or OH

The results show four different pKa values: 3.98, 5.65, 7.76, and 9.49. The pKa-values of 3.98 and 5.65 can be assigned to glutamic and aspartic acid, respectively, the pKa-value of 7.76 can be allocated to lysine or arginine, and the pKa-value of 9.49 cannot be precisely assigned. This pKa-value could be the result of titrated NH_2_- or OH-groups. The hydroxyl group of tyrosine has a theoretical pKa of 10.10 and is amenable to titration. SlfB contains 3.1 mol % of tyrosine, and therefore, is also characterized by potentially free OH-groups. In summary, the amount of modifiable COOH-groups was calculated to be 36.5 mol per mol protein, while the amount of NH_2_-groups was determined to be 12 mol per mol protein when the pKa-value of 9.49 is not considered and 69 mol per mol protein when it is applied to these groups. This means SlfB possesses 36.5 free COOH- and either 12 or 69 free NH_2_-groups theoretically available for modification if the polymeric structure of the protein is stable. It can be assumed that all other COOH- and NH_2_-groups that could be not titrated are blocked via intramolecular and intermolecular interactions of the monomer and the polymer, respectively. A theoretical modification rate of at least 48 mol∙mol_SlfB_^−1^ is therefore possible. SlfB exhibits a p4-symmetry with a length of 13 nm [20] that is consistent with an area of 169 nm². Provided that all available COOH- and NH_2_-groups are modified, then 192 mol of the desired molecules can be linked to an area of 169 nm^2^. However, these calculations do not consider steric hindrance and charge effects. Therefore, it can be expected that the real amount is much lower.

### 2.2. Modification of SlfB with Tryptophan

For a more detailed analysis, in another experiment the amino acid tryptophan was linked to the COOH- and NH_2_-groups of SlfB. This experiment was performed in order to determine a more realistic modification rate. Tryptophan was chosen because of its small size, its minor charge effects due to its neutral character, and its aromatic residual, which allows for the direct quantification of bound tryptophan with UV/VIS-measurements. S-layer proteins were modified with the amino acid tryptophan at their COOH- and NH_2_-groups as described in Section 3.2. These results are shown in the following Table 2.

**Table 2:** Total amount of modified COOH and NH_2_-groups with tryptophan.

Modification of	Modification Rate	Molar Loading (mol_Trp_∙mol_SlfB_^−1^)
NH_2_	26.4% to 56.7%	27.2 to 58.4
COOH	21.2% to 53.4%	26.5 to 66.7

The total amount of theoretically available COOH- and NH_2_-groups is 125 and 103, respectively. The table shows that up to one half of the COOH- or NH_2_-groups of SlfB were modified with tryptophan, which corresponds to a molar load of 67 tryptophan on the COOH- and 58 tryptophan on the NH_2_-groups. This equates to a density of up to 1.58 modifiable COOH-groups per nm^2^ and up to 1.38 modifiable NH_2_-groups per nm^2^. Lower rates were determined with potentiometric titration: 36.5 mol COOH-groups could be titrated and can be claimed to be “available for modification”, corresponding to a density of 0.86 COOH-groups per nm^2^. In case of NH_2_-groups, the number of possible titrated groups was determined to be 12 mol and 69 mol, respectively (Table 1).

It is noted that some carboxylic groups might be deprotonated at pH 3 before starting the titration. These deprotonated groups are not detected by the titration, but they are available for modification, explaining the different values. It is also noted that the complete modification of all COOH- and NH_2_-groups of the protein is not possible. Due to the structure of the protein, interactions of different functional groups of the protein, and steric hindrance, not all groups are accessible for modification with tryptophan. Furthermore, some intramolecular reactions of EDC-activated COOH-groups may occur. However, regarding these aspects and limitations, the obtained modification rate is quite high. In a former publication Weigert *et al*. [21] performed a complex procedure comprising several modification and extraction steps in order to determine the number of free carboxyl groups exposed in the surface of the S-layer lattice of *L. sphaericus* CCM2120. They calculated a charge density of 1.6 carboxyl groups per nm^2^ that is in the range of the values obtained in the present work. However, these researchers investigated the available carboxyl groups of S-layers on cell wall fragments, not of isolated protein polymers or monomers. The methods described in the present work allow a much easier and faster determination of available COOH- as well as of NH_2_-groups.

Experiments in the present study were also performed with protein polymers and monomers. The modification rates of NH_2_-groups were found to be similar for both, whereas the determined amount of modifiable COOH-groups was nearly twice as much for the monomers in comparison to the polymers. These differences can be expected considering that for the polymers the carboxyl groups necessary for exhibiting the polymeric structure are blocked by the binding of Ca^2+^. Conversely, it has been reported that the modification of monomers disturbs the self-assembly properties of the proteins, probably due to the occupation of Ca-binding sites. Therefore, the present study concentrates on S-layers in their polymeric form.

The results showed that at least 20% of the calculated functional groups were modifiable on SlfB. In other work SlfB was modified with FRET-pairs and showed modification rates of only 0.15–0.6 mol_dye_∙mol_SlfB_^−1^ [15]. Although the reported modification rates are not in conflict with those reported here, they prove the influence of molecule size and charge effects on the modification rates. Commercial available fluorescence dyes such as HiLyte^®^ dyes are well-known, highly-negative charged molecules due to their elevated chloride content. Furthermore, the molecular size of the fluorescence dyes is nearly 1000 g∙mol^−1^, five times higher than that of tryptophan with 204 g∙mol^−1^. In a previous study from Küpcü, S-layer proteins were modified with either enzymes or human IgG with the help of EDC [13,17]. In another work, Jahn-Schmid *et al*. conjugated the allergen Bet v1 to different kinds of S-layer products [18]. In these studies similar modification rates with fluorescence dyes could be reached [13].

### 2.3. Inserting SH-Groups to SlfB

Sulfhydryl (SH-) groups are present in cysteine containing proteins and fulfill important functions, e.g., in protein folding or in the active site of enzymes. Unbound or reduced sulfhydryl groups are useful targets for protein conjugation and labeling. Crosslinking via SH-groups is regarded as more selective and precise than labeling via primary amines. However, SlfB, as most bacterial S-layers, does not contain any cysteines, thus prohibiting the use of natural protein-own SH-groups for modification. The lack of SH groups necessitates the introduction of such groups using sulfhydryl-addition reagents such as SATA (*N*-succinimidyl *S*-acetylthioacetate) (Section 3.3.). In this work the suitability of S-layer proteins for such modifications is investigated.

**Figure 1:**
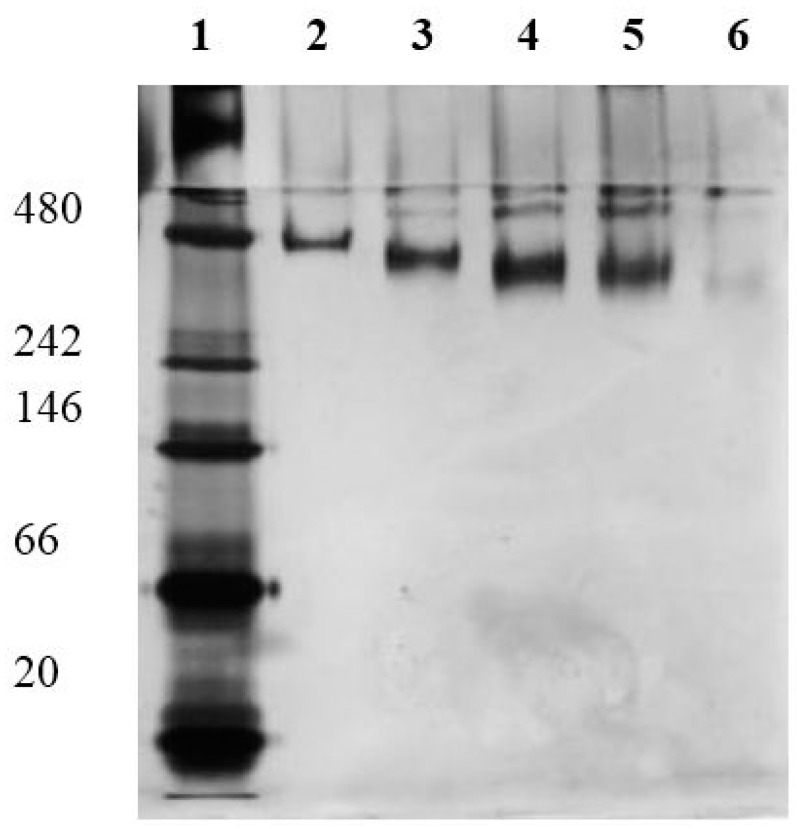
Native PAGE of SlfB modified with different molar excess of SATA; lane 1—molecular weight standard with 480, 242, 66, and 20 kDa; lane 2—native SlfB; lane 3–6—SlfB modified with a 2-, 4-,10-, and 20-fold molar excess of SATA.

The amount of introduced SH-groups was determined by Ellman’s reagent, and a qualitative determination was completed by means of native-PAGE (native polyacrylamide gel electrophoresis) as described in Section 3.3. The results show that the number of introduced sulfhydryl groups increases with increasing amounts of used crosslinker SATA (see Table 3 and Figure 1). A modification rate of 10 mol_SH_∙mol_SlfB_^−1^ could be reached in the case of a 10 and 20 molar excess of SATA. In a native-PAGE the mobility of modified SlfB increases with the amount of generated SH-groups. This is caused by the enrichment of negatively charged groups, and hence, proves the introduction of the new functional SH-group. In Figure 1 the results of the performed native-PAGE are shown. The native SlfB shows an estimated molecular weight of 480 kDa, which corresponds to one S-layer unit cell (p4-symmetry). If this protein is modified with different amounts of SH-groups, the protein shows more mobility resulting in an apparently lower molecular weight. A comparably performed SDS-PAGE did not show any size reducing effects of the protein if modified with SH-groups (data not shown). The higher mobility of SH-modified SlfB proves the successful linking of SH-groups to the protein.

**Table 3:** Modification rate of SlfB modified with SATA; determined by Ellmann’s reagent; * native SlfB was used as a reference.

Molar Excess of SATA to SlfB	Modification Rate (mol_SH_∙mol_SlfB_^−1^)
Reference *	1.5
2-fold	3.8
4-fold	3.7
10-fold	10.0
20-fold	9.5

Principally, both methods are suitable for the determination of SH-groups. However, detection with the help of Ellman’s reagent needs at least 1 mg of protein per sample, whereas in case of native-PAGE only a few micrograms of protein are needed. A disadvantage of the native-PAGE is that only a qualitative check of the success of modification can be performed. For the use of the Ellman’s reagent method, a primary disadvantage is a possibly high inaccurateness as seen in this study. Although SlfB contains no cysteine, with Ellman’s reagent a content of 1.5 mol_SH_∙mol_SlfB_^−1^ could be detected, demonstrating that a high background signal has to be considered and measured modification rates have to be interpreted carefully.

Principally, crosslinking with SATA uses the same amino groups of SlfB as EDC crosslinking with tryptophan. However, the modification rate with SATA was much lower than the modification rate with tryptophan (Table 2). These differences can be explained by the relatively large structure and molecular weight of the crosslinker SATA. As discussed for fluorescent dyes, steric hindrance effects can result in a lower modification rate.

Besides chemical modification, SH-groups can be introduced into proteins also by genetic modifications. Badelt-Lichtblau [22] successfully constructed S-layer proteins with the fusion of a cysteine without influencing the self-assembling properties, thus offering free sulfhydryl groups for the covalent attachment of differently sized molecules as well as for binding of gold nanoparticles. However, generally the introduction of cysteines into proteins, especially into cysteine-lacking proteins, can potentially induce massive structural changes. SH-groups are able to form disulfide bonds and can change the crystallization structure, or even disturb the crystallization process. For this, additional chemicals would be necessary to inhibit the formation of disulfide bonds. Conversely, genetic engineering allows a directed modification of the protein at defined sites, whereas the localization of chemically introduced molecules cannot be controlled. In conclusion, chemical modifications, as performed in this study, are easier and faster to perform than genetic modifications, which presume a deeper knowledge of the protein structure, although the exact location of the modifications within the protein cannot be determined.

In further experiments the SATA modified SlfB proteins were incubated with EDC in order to verify if the NH_2_-groups of SlfB had been successfully blocked by SATA. If so, blocking should prohibit internal crosslinking of S-layer proteins. The internal crosslinking was checked by means of SDS-PAGE as described in Section 3.3.

**Figure 2:**
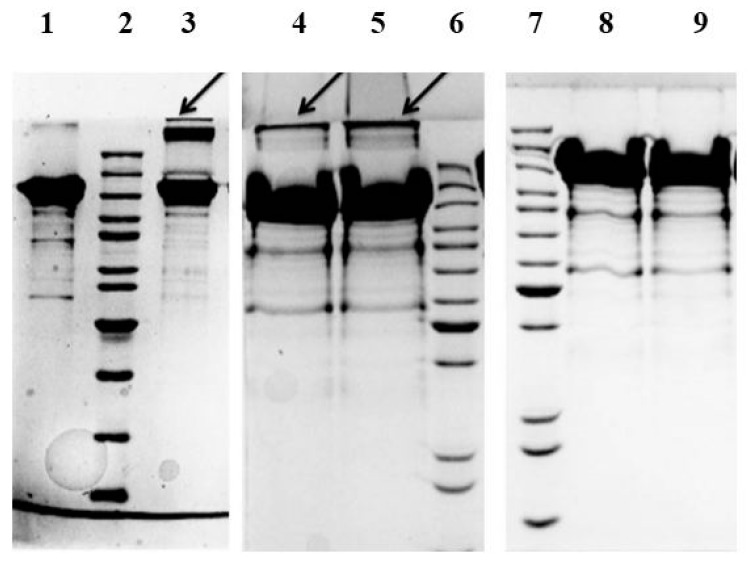
SDS-PAGE of SlfB with different modifications: lane 1—native SlfB; lane 3—native SlfB crosslinked with EDC; lane 4—SlfB modified with an 4-fold excess of SATA and modified with EDC and; lane 5—same protein as in lane 4 and deacetylated; lane 8—SlfB modified with an 20-fold excess of SATA and modified with EDC; lane 9—same protein as in lane 8 and deacetylated; lane 2, 6, and 7—molecular weight standard with 200, 150, 120, 100, 85, 70, 50, 40, 30, 25, and 20 kDa.

In these experiments, using a SATA-excess of up to four times, a crosslinking can still be detected (see arrows Figure 2), which indicates that NH_2_-groups are still available for modification. In contrast, when using a SATA-excess of twenty times, no internal crosslinking could be monitored indicating that all NH_2_-groups are blocked by SATA. Furthermore, a deacetylation with hydroxylamine does not destroy the protein as observed for other proteins [23]. These results show that SATA is a suitable candidate not only for introduction of SH-groups into the protein, but also as a blocking reagent for free NH_2_-groups. The thus SH-modified SlfB proteins are not only attractive for crosslinking with functional molecules, but also for its immobilization on gold surfaces. Currently, we use in our lab polyelectrolytes as an intermediate layer for the attachment of S-layer proteins on surfaces [20]. However, especially for the construction of biosensors that use SPR technologies for detection, a direct attachment to gold surfaces might be more suitable. Another attractive approach for the SH-modified S-layers is the attachment of gold nanoparticles [24]. Such materials can be used for the design of sensory devices or catalytic surfaces.

### 2.4. Biofunctionalization of Surfaces

Due to their self-assembling properties, their arrangement in two-dimensional arrays, and high potential in nanotechnologies, S-layer proteins are attractive as matrices for bioconjugations, thus linking functional molecules to technical surfaces in a highly ordered manner. The use for these purposes implies that the chemical modification does not alter the protein structure, the polymer composition, or the self-assembling properties. In a previous study [15] it was shown that modification of SlfB monomers alter the self-assembling properties seriously. These labeled monomers were not able to form typical S-layer arrays demonstrating a severe change of the protein properties. In contrast, the same study demonstrated that the modification of protein polymers in suspension did not alter the polymeric structure. Modification of these polymers with fluorescent dyes resulted in a FRET, indicating a close proximity of FRET pairs and intact self-assemblies.

**Figure 3:**
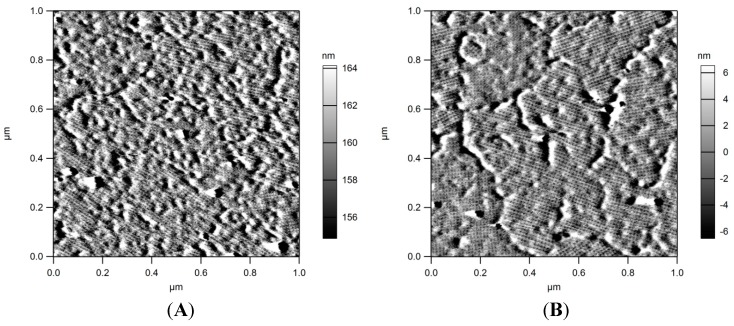
AFM images of SlfB recrystallized on silicon surfaces. (**A**) amplitude image of SlfB prior modification with tryptophan; (**B**) amplitude image of SlfB after crosslinking with tryprophan; S-layer coatings as well as p4-symmetries stay intact.

Despite this success, experiments in our lab demonstrated that the thusly-modified proteins were not applicable for a two-dimensional arrangement on surfaces. Therefore, these constructs are not suitable for a direct application as an agent for the functionalization of surfaces. Instead, another approach was chosen. In a first step, PEI (polyethyleneimine) activated silicon surfaces were coated with SlfB as described in Section 3.5. AFM (Atomic Force Microscopy) analyses proved the formation of the typical S-layer arrays exhibiting p4 symmetry (Figure 3A). In a second step the protein layers were EDC activated and conjugated with tryptophan. AFM analyses proved the intactness of the arrays after this treatment (Figure 3B). These results confirm that the chemical modification of the protein layers do not alter their structure. In a recent work S-layer proteins of *L. sphaericus* JG-B53 were recrystallized on PEI modified silicon surfaces and layer thickness was determined by AFM and QCM-D. The formed layers exhibited a thickness of about 12.9 nm indicating the formation of monolayers [12].

It has to be emphasized that assembly of SlfB on surfaces almost certainly decreases the number of modifiable COOH- and NH_2_-groups. Properties and accessibility of the proteins in suspension naturally differ from those of the proteins placed on surfaces. On the other hand, a reliable determination of available modifiable groups on protein coatings is in view of low protein amounts (about 5 µg∙cm^−2^) not possible with current methods. However, the previous experiments showed that in suspensions numerous COOH- and NH_2_-groups of the proteins could be modified. These data allow a rough estimation of the modifiability of the proteins. It can be expected that a reasonable amount of these groups are still accessible after coating.

There are several studies that describe the immobilization of S-layer proteins followed by chemical modification with enzymes, antibodies, or antigens, thus producing functionalized surfaces. These approaches used cell wall preparations, peptidoglycan, liposomes, and ultrafiltration membranes [13,17,18,25,26] as immobilization matrices. Further, there are numerous examples of immobilized genetically engineered fusion proteins on different surfaces (listed in [1]). In contrast to these works, the present work describes a method that allows a fast and reliable patterning of various kinds of technical surfaces and an easy immobilization of organic molecules on the these nanostructured surfaces. In a current study [27] S-layer proteins were modified with thrombin-binding or ofloxacin-binding aptamers (short oligonucleotides). The thus immobilized aptamers kept their functionality, thus proving the suitability of the presented bio-functionalization method.

## 3. Experimental Section 

### 3.1. Potentiometric Titration of SlfB

Potentiometric titration experiments were carried out under a nitrogen atmosphere at 25 °C. About 60 mg SlfB protein were suspended in 30 mL carbonate-free deionized water. The ionic strength was adjusted to 0.1 M (NaCl). Titration was started at pH 3 (adjusted with HCl) and carried out up to pH 10 with carbonate-free 0.01 M NaOH (Titrisol, Merck, Darmstadt, Germany).

The 0.01 M titration solution was adjusted to I = 0.1 M with NaCl. The pH was measured with a BlueLine 16 pH electrode (Schott, Mainz, Germany). The electrode was calibrated for each experiment with NIST/PTB standard buffers (pH 4.008, 6.865, and 9.001, Schott). The minimum drift was set to 0.5 mV∙min^−1^, and a delay time of at least 60 s at each titration point was maintained before measuring the pH value. The dynamic titration procedure was performed with an automatic titrator (TitroLine alpha plus, Schott), monitored by the accompanied software (TitriSoft 2.11, Schott), and analyzed with the Hyperquad 2006 software [28].

### 3.2. Chemical Modification of SlfB with the Amino Acid Tryptophan

The chemical modification of COOH- and NH_2_-groups of the S-layer proteins was performed using the reagent EDC, which initiates the formation of a peptide bond between a COOH- and an NH_2_-group by activating the COOH-group of the protein or amino acid. For the chemical modifications of the COOH-, NH_2_-, or both groups of the protein, tryptophan was used as the modifying agent. If both functional groups of the proteins were modified, the modification was performed in two steps. In case of NH_2_-groups, tryptophan was activated with EDC. In case of COOH-groups, EDC was first added to the S-layer proteins, and afterwards tryptophan was added in 100 or 200 molar excess to the protein. 

SlfB were used in their polymer form and were diluted in 100 mM CaCl_2_ to a concentration of 5 mg∙mL^−1^ at a pH of 6.5. EDC was added to S-layer proteins or tryptophan as solid powder. Two different approaches were used: modification of S-layer proteins NH_2_-groups by using EDC-activated tryptophan and modification of S-layer proteins COOH-group by activation of COOH-groups of the protein with EDC.

For the activation of tryptophan, tryptophan was diluted in deionized water to a concentration of 5 mmol∙L^−1^, and EDC was added to tryptophan in a molar ratio of 2:3 with respect to tryptophan followed by an incubation for 30 min at room temperature. Afterwards, activated tryptophan was added to the protein in 100 or 200 molar excess to the SlfB, and samples were incubated for 2 h at room temperature.

For the activation of S-layer proteins, S-layer proteins were incubated for 30 min with EDC in a molar excess of 5000 with respect to the SlfB. After incubation tryptophan was added with a molar excess of 100 or 200. The activated S-layer protein and solution was incubated for 2 h at room temperature.

Excess tryptophan was removed by ultrafiltration. For this, 500 µL of the samples were transferred to ultrafiltration membranes (50 kDa cut off centricons, Merck Millipore, Darmstadt, Germany), and solutions were centrifuged at 6000× *g* for 40 min at 20 °C. The filtrate contained unbound tryptophan. The content was investigated by UV/Vis-measurements at 278 nm, and the amount of unbound tryptophan was determined with the using a calibration curve and Beer-Lamberts-law.

In case of both functional groups of the S-layer proteins modified with tryptophan, unbound tryptophan of the first modification step was removed by dialysis in the presence of 100 mM CaCl_2_. The dialyzed S-layer proteins were further modified with tryptophan at their COOH-group as described above.

### 3.3. Inserting SH-Groups to SlfB and Internal Crosslinking of SlfB with EDC

#### 3.3.1. Chemical Modification of S-Layer Protein SlfB with SATA

For the insertion of SH-groups, the reagent *N*-succinimidyl *S*-acetylthioacetate (SATA, Thermo Fischer Scientific, city, country) was used. This reagent contains a succimidyl at one end to be attached to protein NH_2_-groups and a protected SH-group, namely an acetylthiopropionate, on the other end. If SATA is attached to the protein the SH-groups will not build disulfide bonds and the native protein structure is protected. To get free SH-groups, hydroxylamine can be used as deacetylation reagent.

SATA was linked to SlfB by a procedure described by the manufacturer. SlfB was suspended as polymer in 0.1 M HEPES (4-(2-hydroxyethyl)-1-piperazineethanesulfonic acid) at pH 7.2 with a concentration of 11 g∙L^−1^. SATA was dissolved in dimethyl sulfoxide (DMSO) to a final concentration of 17 nmol∙mL^−1^. For the optimization of modification, different molar excesses ranging from 5 to 20 of SATA to SlfB were used. Protein suspensions were cooled on ice for the coupling reaction in order to stabilize the proteins when adding DMSO with SATA. SATA was added to the proteins until the desired molar excess was achieved. The proteins had a final concentration of 10 g∙L^−1^ and the DMSO concentration was 10% (*v*/*v*). The reaction took place over night at 4 °C. After the binding of SATA to the protein, excess SATA and DMSO were removed by centrifugation. SlfB was centrifuged at 12,400*× g* for 60 min and 4 °C and washed two times with 10 mM CaCl_2_.

#### 3.3.2. Determination of Total Amount of SH-Groups

Ellman’s reagent was used for the determination of SH-groups. First, the acetylated SH-groups were deacetylated with hydroxylamine. For this, the protein samples were incubated 2 h with 2.5% hydroxylamine and 10 mM TCEP (tris(2-carboxyethyl)phosphine) at room temperature. TCEP inhibits the building of disulfide bonds and does not interfere with Ellman’s reagent. After deacetylation, 100 µL of the protein sample, which represents 1 mg protein, were applied to Eppendorf reaction tubes, and 1 mL of 0.1 M Na_3_PO_4_ pH 8 with 1 mM EDTA (ethylenediaminetetraacetic acid) was added to the protein solution and mixed. Afterwards, 20 µL of a 4 g∙L^−1^ Ellman’s reagent was added to the sample and incubated for 15 min at room temperature. Afterwards, UV/Vis-measurements were performed at 412 nm and absorption was determined. Using a calibration curve, which was established with cysteine, the total amount of free SH-groups in the protein was determined.

Native-PAGE was performed for a qualitative detection of SH-groups. For the native-PAGE, 0.5 µL of the deacetylated protein solution were mixed with 5 µL loading buffer (10% glycine and 0.01% Ponceau S) and 20 µL lysis buffer (50 mM NaCl, 5 mM 6-aminohexanoic acid, 50 mM imidazole) and incubated for 10 min at room temperature. Afterwards, the samples were applied to the gels. The protein ladder from Life Technologies GmbH (Darmstadt, Germany) was used for the estimation of the molecular weight of the proteins and ranged from 20 to 1200 kDa. The gels were stained with Coomassie Brillant Blue G250.

#### 3.3.3. Internal Crosslinking of SH-Modified SlfB with EDC and Deacetylation of Modified SlfB to Get Free SH-Groups

S-layer proteins SlfB, which were modified with different molar excess of SATA, and native SlfB were internally crosslinked with EDC. EDC initiates a covalent peptide bond between COOH- and NH_2_-groups. For the experiments, 1 mg of SATA-modified SlfB were incubated with 20 mM EDC. For this, 10 µL of 200 mM EDC were added to 90 µL protein solution and incubated at room temperature for 2 h. 50 µL of each protein sample were deacetylated with hydroxylamine as described in Section 3.3.2. 10 µg of each protein solution (acetylated and deacetylated form) was then investigated by SDS-PAGE. SDS-PAGE was performed as described in [29], and 10% polyacrylamide gels were used. Proteins were then stained with Coomassie Brillant Blue G250. A standard protein ladder from Thermo scientific was used with a range from 200 to 20 kDa.

### 3.4. Preparation of the S-Layer Protein SlfB

S-layer proteins were used from the natural isolate *Lysinibacillus sphaericus* JG-A12. Cells were cultivated in a 50 L biorecator for 5–6 h in nutrient broth media at 30 °C with an oxygen supply of 30%. Cells were harvested at the end of their exponential growth phase and rinsed with standard buffer solution (50 mM Tris, 1 mM MgCl_2_, 10 mM CaCl_2_, 3 mM NaN_3_, pH 7.5). The isolation of the S-layer proteins was performed in the following three step procedure: cell fracture by high pressure homogenization, removal of plasma membrane and peptidoglycan by Triton X-100 and lysozyme, and dissolving of polymer structure with guanidine hydrochloride and self-assembly by dialysis against crystallization buffer (1.5 mM Tris, 10 mM CaCl_2_, pH 8). Purification of S-layer proteins was monitored by SDS-PAGE. Detailed protocols for every step are described elsewhere [12]. The isolated S-layer proteins were lyophilized and stored at 4 °C until further use. Protein polymers can be generated by dissolving the lyophilized proteins in crystallization buffer.

### 3.5. Coating of Surfaces and Bioconjugation

First, the surfaces of silicon dioxide wafers (5 mm × 5 mm) were cleaned using the RCA method [30]. Second, the surfaces were covered with a layer of the positively charged polyelectrolyte (PEL) polyethyleneimine (PEI, MW 25,000, Sigma-Aldrich, St. Louis, MO, USA). PEI was solved in deionized water to a concentration of 3 g∙L^−1^. The silicon wafers were incubated with each PEI for 1 h. Afterwards the system was rinsed with deionized water to remove loosely bound PEI and incubated overnight in deionized water before crystallization of the S-layer. Therefore, lyophilized SlfB dissolved in 6 M guanidine hydrochloride was added to crystallization buffer to a final protein concentration of 0.1 g∙L^−1^ for recrystallization on the PEI-layer. After assembly of the S-layer on the PEL coated silicon surface tryptophan modification via the COOH-groups of SlfB monolayer (see Section 3.2.) using excess EDC (10 mM) for activation was conducted in presence of 10 mM CaCl_2_.

The existence of the SlfB monolayer on silicon wafer surface before and after tryptophan modification was visualized with atomic force microscopy (AFM) using a MFP3D Bio instrument (Asylum Research, Santa Barbara, CA, USA). Measurements were conducted as described elsewhere [20].

## 4. Conclusions

The aim of the present work was the investigation of the S-layer protein of *Lysinibacillus sphaericus* JG-A12 regarding its suitability for performing bioconjugations and coating of surfaces, thus creating new possibilities and materials for the functionalization of surfaces. Using potentiometric titration the total amount of available and therefore, potentially modifiable COOH-groups and NH_2_-groups were determined as 36.5 mol and 12–67 mol per mol protein, respectively. The modification of these functional groups with tryptophan, as small molecule with little steric hindrance effects, was investigated, and the amount of bound tryptophan supports the yielded results by potentiometric titration. Amounts of over 50 mol of COOH- or NH_2_-groups per mol protein of SlfB could be modified with tryptophan without destroying the polymeric structure of the protein. 

Additionally, SH-groups were introduced to the protein with the crosslinker SATA. This resulted in an insertion of a maximum of 10 mol SH-groups per mol S-layer protein. A subsequent crosslinking of the S-layer protein with EDC showed that the modification with SATA worked as a successful blocking reagent for modifiable NH_2_-groups and inhibits the internal crosslinking of the S-layer protein when COOH-groups are modified with EDC.

Furthermore, silicon substrates were coated with S-layer protein arrays, and proteins were modified with tryptophan. AFM images demonstrated that this modification did not alter the array structure. Although in these experiments the total amount of modified groups could not be determined in this approach, it can be expected from the results of potentiometric titration that a reasonable number of COOH-groups and NH_2_-groups were still available for modification.

In summary, this paper demonstrates the chemical modification of S-layer proteins for the design of biofunctionalized surfaces for a diversity of applications, such as biosensors, (bio)catalytic surfaces, or filter materials. Several studies described the introduction of functional groups using cross-linking methods [13,17,18,25,26]. However, these studies did not investigate the potential modification rate in detail, nor described the amount of introduced functional groups. The high modification rate for small molecules like tryptophan promises a high pack density of functional molecules on the surface, and the nanostructured surface of the S-layer arrays guarantees a highly ordered arrangement of the attached molecules.
